# Biological Control of Plant-Parasitic Nematodes by Filamentous Fungi Inducers of Resistance: *Trichoderma*, Mycorrhizal and Endophytic Fungi

**DOI:** 10.3389/fmicb.2020.00992

**Published:** 2020-05-25

**Authors:** Jorge Poveda, Patricia Abril-Urias, Carolina Escobar

**Affiliations:** ^1^Spanish-Portuguese Institute for Agricultural Research (CIALE), University of Salamanca, Salamanca, Spain; ^2^Biological Mission of Galicia (MBG-CSIC), Pontevedra, Spain; ^3^Facultad de Ciencias Ambientales y Bioquímica, Área de Fisiología Vegetal, Universidad de Castilla-La Mancha, Toledo, Spain; ^4^International Research Organization for Advanced Science and Technology, Kumamoto University, Kumamoto, Japan

**Keywords:** plant-parasitic-nematodes, *Trichoderma*, mycorrhizal fungi, endophytic fungi, biocontrol, plant systemic resistance

## Abstract

Plant-parasitic-nematodes represent a major threat to the agricultural production of different crops worldwide. Due to the high toxicity of chemical nematicides, it is necessary to develop new control strategies against nematodes. In this respect, filamentous fungi can be an interesting biocontrol alternative. The genus *Trichoderma*, mycorrhizal and endophytic fungi are the main groups of filamentous fungi studied and used as biological control agents (BCAs) against nematodes as resistance inducers. They are able to reduce the damage caused by plant-parasitic nematodes directly by parasitism, antibiosis, paralysis and by the production of lytic enzymes. But they also minimize harm by space and resource-competition, by providing higher nutrient and water uptake to the plant, or by modifying the root morphology, and/or rhizosphere interactions, that constitutes an advantage for the plant-growth. Besides, filamentous fungi are able to induce resistance against nematodes by activating hormone-mediated (salicylic and jasmonic acid, strigolactones among others) plant-defense mechanisms. Additionally, the alteration of the transport of chemical defense components through the plant or the synthesis of plant secondary metabolites and different enzymes can also contribute to enhancing plant defenses. Therefore, the use of filamentous fungi of the mentioned groups as BCAs is a promising durable biocontrol strategy in agriculture against plant-parasitic nematodes.

## Introduction

Over 4100 species of plant-parasitic nematodes were described to date, among which, a restricted group of genera is considered as major plant-pathogens, whereas others are specific to a more limited range of crops, both causing a high impact to economically important crops. Damage caused by plant nematodes has been estimated as a projected yield loss of 12.3% ($157 billion) worldwide (Singh et al., 2015), more significant damage comparing to invasive insects (around US$70 billion; Bradshaw et al., 2016). The full extent of worldwide nematode damage is likely to be underestimated, since growers are often unaware of their presence because the symptoms caused in the plant are often non-specific, making difficult to attribute crop losses to nematode damage (Jones et al., 2013; Siddique and Grundler, 2018). Additional losses could be related to food quality and visual imperfections associated with infection symptoms (Palomares-Rius et al., 2017).

These nematodes are usually small soil-borne pathogens that can feed on all plant parts (including roots, stems, leaves, flowers and seeds), although most species feed on roots. They need a protrusible stylet for feeding that they use to penetrate the plant cells. The stylet is connected to three to five pharyngeal glands that produce effector molecules, which are often secreted, facilitating penetration, internal migration, and parasitism (Jones et al., 2013; Mejias et al., 2019). Based on their feeding habitats, plant-parasitic nematodes can be broadly categorized as either ectoparasitic or endoparasitic. However, the most important nematodes in terms of crop losses are sedentary endoparasites, the root-knot (*Meloidogyne* spp.) and cyst nematodes (*Heterodera* and *Globodera* spp.; Jones et al., 2013). Nowadays, there is an increasing need to supply more food to the growing population (FAO, 2017); therefore, the control of plant-parasitic nematodes is also a growing global demand.

Given the high economic impact caused by parasitic nematodes, a large number of strategies have been developed for nematode-control in agriculture, including the use of chemical nematicides. However, the need to deploy effective nematode resistance is intensified with the loss of pesticides due to EU regulations (EC No1107/2009) as they are harmful to human health and contaminant for the environment (Zhang et al., 2014, 2017). Strategies associated with biocontrol are proposed as a much safer alternative and highly practicable for plant-parasitic nematodes management. The term biological control (or biocontrol) applies to the use of living organisms to suppress the population density or impact to a specific pest organism, making it less abundant or less damaging than it would otherwise be. Specifically, biological control of nematodes is defined as the regulation of nematode populations and/or a reduction in nematode damage through the action of organisms antagonistic to them, which occur naturally or through the manipulation of the environment or the introduction of antagonists. The organism that suppresses the pathogen is referred to as the biological control agent (BCA) that can interact directly with the pathogen where we include antagonism (antibiosis and competition for nutrients or space among others) or interact indirectly with the pathogen through the host-plant as for example inducing plant-resistance (systemic acquired resistance or SAR and induced systemic resistance or ISR; Pal and Gardener, 2006; Stirling, 2018; Xiang et al., 2018). Nowadays, a wide variety of organisms are known to act as BCAs against plant-parasitic nematodes such as fungi, bacteria, viruses, protists, nematode antagonists and other invertebrates. BCAs, physical methods such as solarization and fallowing or cultural practices as crop rotation were proved in most cases viable applied in combination and/or with reduced doses of chemicals in a scenario of integrated pest management (Khan and Kim, 2007; D’Addabbo et al., 2019). Biocontrol of plant-parasitic nematodes using predatory nematodes dates back to the early 20th century; nevertheless, their potential has only begun to be studied in recent years. Apart from acting as BCAs against plant-parasitic nematodes, these nematodes play a main role in stimulating cycling of plant nutrients, which allow plants to defend themselves more effectively against the attack of pathogens. There are many examples described in the literature, among which we find for example *Odontopharynx longicaudata*, effective against *M. incognita* and *M. javanica* (among others) and *M. gaugleri*, effective against *Heterodera oryzae* and *M. incognita* (among others; Khan and Kim, 2007).

Interestingly, plant growth-promoting rhizobacterias (PGPRs) enhance plant growth by colonizing the plant root system, but some PGPR showed also nematicidal activity against plant-parasitic nematodes. One clear example is the secondary metabolites produced by *Pseudomonas fluorescens* CHA0 that induce mortality of nematode eggs and second-stage infective juveniles (J2s; Siddiqui and Shaukat, 2003). In a study conducted by Zhao et al. (2018), from 860 strains of bacteria collected from the rhizosphere, 5 showed high efficacy as BCAs against *Meloidogyne javanica*, i.e., *Bacillus cereus, B. subtilis, Pseudomonas putida, P. fluorescens*, and *Serratia proteamaculans.* Hence, PGPRs, in addition to benefit plants growth, have great potential through direct interaction against pathogens such as nematodes.

Plants also have a series of innate defensive strategies to defend themselves from the attack of pests and pathogens in a targeted manner. Once the attack occurred, the plants recognize non-specific molecules of the cell wall of the microorganisms [pathogen- and/or Microbial-Associated Molecular Patterns (PAMPs/MAMPs)], the oral secretions of the herbivores [herbivore-associated molecular patterns (HAMPs)] or signs of cell-plant damage [Damage-Associated Molecular Patterns (DAMPs)], by cell surface pattern recognition receptors (PRRs) (Mithöfer and Boland, 2008; Ramírez-Prado et al., 2018; Hou et al., 2019). Nematode-Associated Molecular Patterns (NAMPs) and its associated receptors have been scarcely described (reviewed in Ali et al., 2018). However, it is generally accepted that during the plant-pathogen interactions, the detection of PAMPs and DAMPs by PRRs triggers a complex network of intracellular signaling cascades leading to defense responses known as PAMP-triggered immunity (PTI). It involves the induction of plant responses coordinated by stress-hormones such as salicylic acid (SA), mostly associated to biotrophic pathogens, and jasmonic acid (JA), and ethylene (ET) against necrotrophic pathogens and herbivores. Additionally, the pathogens generate a diverse repertoire of effector proteins that avoid PTI, a phenomenon known as effector-triggered susceptibility (ETS), against which plants produce specific effector receptors that activate a specific effector-triggered immunity (ETI) (Shigenaga et al., 2017; Ramírez-Prado et al., 2018).

The plant innate immune system comprises local and systemic defense responses. After the attack of a biotroph pathogen and the occurrence of a programmed cell death response in plants, called hypersensitive response (HR), a broad-spectrum immunity to reinfection through the whole plant body, called systemic acquired resistance (SAR) is activated in the plant. SAR signaling is mainly mediated by SA derived compounds, such as methyl-SA (MeSA). Similarly, numerous beneficial microorganisms are capable of inducing in the plant what is known as induced systemic resistance (ISR) against necrotrophic pathogens and herbivores. ISR is regulated mainly by JA/ethylene (ET) signaling, but dependence on SA signaling has also been reported (Van Wees et al., 2008; Martinez-Medina et al., 2016). Systemic resistance mediated by JA is also induced by wounding and is referred to as wound-induced resistance. This type of systemic resistance against the recognition of molecular patterns promotes an improved state of defenses in the plant, which causes the plant to respond much more quickly and effectively against a biotic attack. This process is known as defense priming, sensitization or trained immunity (Reimer-Michalski and Conrath, 2016; Mauch-Mani et al., 2017; Bürger and Chory, 2019).

While fungal pathogens have detrimental effects on plant physiology, mutualistic fungi augment host defense responses to pathogens and herbivores. Fungal partners secrete bioactive molecules such as small peptide effectors, enzymes and secondary metabolites that facilitate colonization and contribute to both symbiotic and defense against pathogenic relationships (Zeilinger et al., 2015). *Trichoderma* species, arbuscular mycorrhizas, ectomycorrhizas, endophytes, yeasts, and avirulent/hypovirulent strains of certain pathogens are among the main beneficial fungi with biocontrol capacity, also by induction of ISR (Martínez-Medina et al., 2013; Ghorbanpour et al., 2018). In this context, we will focus this review in those studies of the genus *Trichoderma*, mycorrhizal and endophytic fungi as BCAs for nematode control.

### General Characteristics of Filamentous Fungi From the Genus *Trichoderma*, Mycorrhizal and Endophytic Fungi as BCAs

#### Genus *Trichoderma*

The *Trichoderma* genus includes a group of anamorphic filamentous fungi whose teleomorphic status has been described in several species, within the *Hypocrea* genus (Martínez et al., 2015). *Trichoderma* includes fungi characterized by rapid growth that produce large amounts of conidia whose pigmentation can vary from dark to light green. They grow naturally in different habitats in a wide range of climatic zones from polar to equatorial latitudes; hence, they are the most isolated fungi from the soil (Vinale et al., 2008; Martínez et al., 2015). The species of the genus *Trichoderma* are ubiquitous colonizers of cellulosic materials and, therefore, can be found wherever there is decaying plant material available, as well as in the plant rhizosphere. However, there are also species specifically limited to some geographic areas (López-Bucio et al., 2015).

A comparative analysis of the genomes of three species of the genus *Trichoderma*, *T. reesei*, *T. atroviride*, and *T. virens*, has determined that mycoparasitism is the ancestral way of life of this genus and that, subsequently, the rhizosphere colonization evolved. In this respect, the presence of pathogens in the soil and the exudates from the plant-roots favored a shift toward a more generalist way of life (Kubicek et al., 2011, 2019). The comparative genomics of these three specific species suggests that the saprotrophic species *T. reesei* has a smaller set of genes related to mycoparasitism, thus these genes should have subsequently been lost in *T. reesei* and other species (Kubicek et al., 2011).

*Trichoderma* acts as a symbiont capable of colonizing the roots (without reaching the vascular bundle, limited only to the outermost layers) establishing a complex molecular dialogue with the plant-host. This relationship, together with its easy adaptation to various climatic and edaphic conditions, and its growth speed, give this genus an advantage over many other filamentous fungi and make them excellent candidates as BCAs. Currently, several mechanisms of action of *Trichoderma* are recognized as BCA: mycoparasitism, antibiosis, competition with the pathogen, promotion of plant growth, enhanced plant-tolerance against abiotic stresses and stimulation of its defenses against pathogens (Hermosa et al., 2012; Poveda, 2020).

#### Mycorrhizal Fungi

Mainly used as biofertilizers, mycorrhizal fungi are obligate symbionts of the roots of 97% of the vascular plants. Ectomycorrhizal fungi (ECMF) and arbuscular mycorrhizal fungi (AMF) are the main groups and of great interest in forestry and agronomy, since the AMF are capable of forming symbiotic interactions with 80% of the plant species (Berruti et al., 2016; Ferlian et al., 2018). ECMF mainly include the filum Basidiomycota, some Ascomycota, and very few Zygomycota, capable of colonizing the intercellular space of roots from forest species and forming the so-called Hartig network (Domínguez-Núñez and Albanesi, 2019). Whereas, the AMF, belong to Glomeromycota and establish a root symbiosis intracellularly where they develop specialized structures called arbuscules in intimate contact with the plant cells cytosol (Berruti et al., 2016).

At present, molecular and paleo-biological studies have shown how the origin of AMF and of terrestrial plants occurred simultaneously over time (about 470 million years ago) from epiphytic fungi that grew on the surface of the first vascular plants. They were also necessary for the success of plant terrestrial colonization (Feijen et al., 2018; Rimington et al., 2018; Strullu-Derrien et al., 2018). Thus, ECMF emerged evolutionarily after AMF, once the plants had already colonized the earth (Hoeksema et al., 2018).

Development of a mycorrhizal symbiosis requires continuous signals exchange between the two symbionts, which triggers coordinated differentiation of both partners. Moreover, the intimate interaction of the arbuscules within the root cells requires also a partial suppression of plant defense responses (Liao et al., 2018). Mycorrhizal hyphae are able to colonize places in the soil where plant roots could never reach; besides, hyphae have the ability to absorb nutrients due to active transporters. The fungus can contribute mostly to the supply of phosphorus to the plant, but also other nutrients with low mobility, such as ammonium, potassium, copper, iron, sulfur, molybdenum or zinc. In response, the plant must provide carbohydrates to the fungus, which meet their needs, although it does not have a negative impact on the plant, due to photosynthetic compensation with the fungal supply of nutrients and reduced root development (Berruti et al., 2016; Chen et al., 2018). Moreover, it is widely believed that the inoculation of mycorrhizal fungi provides tolerance to host plants against various stresses like heat, salinity, drought, metals and extremes of temperature (Begum et al., 2019).

The similarities between the fungi structural organs used for nutrient uptake between the biotrophic phytopathogenic fungi (haustoria form) and the AMF (arbuscules form), together with the establishment of a long-term relationship with their host, lead to an incorrect response by the plant. First, the plant recognizes PAMPs/MAMPs type molecular patterns (e.g., chitin), which triggers a generalist defensive response of the PTI or MAMP-triggered immunity type. Subsequently, the recognition of effectors released by the fungus in response to plant defenses activates a more specific defensive response (ETI). This part of the plant responses establishes the main difference between the pathogenic fungus and the symbiont fungi (Jacott et al., 2017). The continuous recognition of the AMF within the colonized cells causes the activation of a JA-mediated SAR through the plant termed as mycorrhizal induced resistance (MIR; Pozo and Azcón-Aguilar, 2007). Due to this mechanism, an increase in the systemic accumulation of different defense compounds, such as phytoalexins, suberin, callose, lignin, ROS, phenolic compounds, terpenoids, PR proteins and sulfur amino acids, was observed in mycorrhizal plants. Accordingly, enhanced enzymatic activity of chitinases, glucanases, proteases, ribonucleases, PALs, chalcone synthases and peroxidases was also detected (Hohmann and Messmer, 2017; Jacott et al., 2017; Hill et al., 2018).

#### Endophytic Fungi

The classic definition of plant endophytes refers to those microorganisms that can be isolated from plant tissues once they have been superficially disinfected. They also do not cause visible damage to plants. This group of microorganisms includes bacterial, archaeal, fungal and protist taxa. Among them, the fungal group play an important role in ecosystems, protecting plants against biotic and abiotic stresses (Lugtenberg et al., 2016; Yan et al., 2019). Numerous studies have indicated that these fungi have a wide biotechnological potential because they biosynthesize molecules, such as enzymes, that can be used as biocontrol and plant-growth-promoting agents as well as in bioremediation, biodegradation or biotransformation (Zheng et al., 2016). In turn, endophytic fungi also have a crucial role in nutrients cycling because, once the plant dies, they behave like saprophytes living from plant tissues (Saikkonen et al., 2015).

Although, endophytic fungi are found in a great variety of plants and ecosystems, there is a strong influence of the local environment in determining endophytic communities; hence, individual endophytes show a different niche occupancy (Glynou et al., 2016), accordingly, they are likely to be affected by future climate change (Giauque and Hawkes, 2016). Despite this geographical distribution, the systemic colonization of plant tissues by endophytic fungi is widely regulated by strong antagonism between the different species (which may run into hundreds), confirming that the systemic effects observed in plants by endophytic fungi are mostly due to chemical movement (Yan et al., 2015).

As mentioned before, endophytic fungi are capable of producing plant-associated metabolites and their analogs, bioactive compounds with diverse biotechnological applications, which have been extensively reviewed by numerous authors (Ludwig-Müller, 2015; Nisa et al., 2015; Saikkonen et al., 2016; Vasundhara et al., 2016; Yan et al., 2018). Among them, compounds with cytotoxic capacity against cancer cells and other diseases, such as taxol, podophyllotoxin, camptothecin, and vinca alkaloids have also been found (Gouda et al., 2016; Uzma et al., 2018). But, they are also capable of producing various mixtures of carbon-based compounds, which are known as volatile organic compounds (VOCs) with a direct application as pesticides (Kaddes et al., 2019).

The use of BCAs from endophytic fungi is a growing area of research as shown from numerous reviews on this topic (Chadha et al., 2015; Card et al., 2016; De Silva et al., 2019; Rabiey et al., 2019). The main mechanisms of direct interaction described include the production of lytic enzymes and/or secondary metabolites (antibiosis) and competition.

Endophytic fungi are also able to induce in plants SAR and ISR against the attack of pests and/or pathogens, but they also need to suppress, at least partially, the defenses of the plants to be able to colonize their tissues (Busby et al., 2016). *Epichloë* genus includes important endophytic fungi of grasses widely studied for their ability to protect the plants due to the synthesis of different alkaloids. Besides, these fungi enhance plant immunity against chewing insects by promoting endogenous defense responses mediated by the JA pathway (Bastias et al., 2017) and against leaf-fungi as *Bipolaris sorokiniana* in perennial ryegrass (*Lolium perenne*) by *Epichloë festucae* var. *lolii* (Li et al., 2018). JA-mediated defenses were also observed after the application of the endophytic fungus *Chaetomium cochlioides* on leaves of *Cirsium arvense* (Hartley et al., 2015). Furthermore, the application of *Penicillium citrinum* and *Aspergillus terreus* in sunflower against the stem rot caused by the fungus *Sclerotium rolfsii* increased the levels of SA and JA in the plant (Waqas et al., 2015).

### Direct Interactions of Filamentous Fungi From the Genus *Trichoderma*, Mycorrhizal and Endophytic Fungi for Nematode Control

#### Genus *Trichoderma*

Sahebani and Hadavi (2008) reported that under greenhouse conditions, the inoculation of tomato seeds with *T. harzianum* significantly reduced the level of disease caused by the nematode *Meloidogyne javanica*; affecting their establishment, development and reproduction (i.e., parameters as the number of galls per plant, the number of egg masses per plant, as well as the number of eggs in each mass). Additionally, they observed a significant reduction in egg hatching, thus demonstrating that this species of the genus *Trichoderma* has significant potential as BCA against this plant parasite. Similarly, root colonization by *T. harzianum* impeded nematode performance locally at multiple stages of the parasitism: invasion, galling and reproduction in tomato (Martínez-Medina et al., 2017b).

Moreover, the effect of both the suspension culture and the exudates produced by different species of *Trichoderma*, in the control of *M. incognita* on tomato plants has been evaluated. The metabolites produced by the fungus, when growing in liquid culture, have a direct and very effective effect on *M. incognita*, since it significantly decreased eggs hatching and increased the mortality of the J2. Soil application of culture suspension (containing the fungus spores) affected more negatively to the population of J2s, in addition to increasing plant growth more effectively than fungus exudates (Khan et al., 2018). In both cases, the species with the best results in nematode control was *T. harzianum*. This study confirms the potential as BCAs against this nematode described previously by Sahebani and Hadavi (2008), but also the potential of other species of this genus as *T. viride*.

Furthermore, approximately 1 month after the penetration of the J2 from the root-knot nematodes (*Meloidogyne* spp.), the eggs are laid surrounded by a gelatinous matrix that constitutes an egg-mass secreted by the female, which is considered as a defensive envelope that protects the eggs against microorganisms allowing eggs survival on the soil. According to Sharon et al. (2007), the gelatinous matrix plays a key role in the process of *Trichoderma* conidial union to the nematode *M. javanica*, thus explaining the parasitism resulting from some species of this genus of fungi. However, it cannot be generalized for all species since, e.g., the growth of *T. harzianum* used in this study was inhibited by this matrix, thus not being effective in biocontrol against *M. javanica*.

The genus *Trichoderma* has an important potential as BCA, not only against root-knot nematodes but also against cyst-forming nematodes by direct parasitism of both eggs and larvae. It increase the level of extracellular enzymes like chitinase and protease, which allow the penetration of the fungus into the eggs by directly affecting very abundant structural components of the eggshell, thus reducing the number of eggs capable of hatching and therefore, the number of infective J2. Specifically, *T. longibrachiatum* has a strong inhibitory effect on the hatching of cysts produced by *Heterodera avenae*, since the spores completely cover the surface of these structures, causing their destruction, which is probably due to the production of enzymes (e.g., chitinases) that caused physiological alterations to the cysts (Zhang et al., 2014). *T. longibrachiatum* also showed an effect on females and on the development of both eggs and J2s of *H. avenae* (Zhang et al., 2017), hence *T. longibrachiatum* can be used as a BCA for the management of *H. avenae* in selected crop species. Within the cyst-forming nematodes, the specie *Globodera pallida* has also a high agronomic impact. Contina et al. (2017) used a strain of *T. harzianum* labeled with GFP as a biomarker to study the fungus-nematode interaction, confirming the reduced infection and reproduction of the nematode. The fungus was capable of negatively affecting both cysts and J2s of *G. pallida*, however, no effect was observed on the eggs. Besides, *T. harzianum* established hyphal colonization in the rhizoplane and the rhizosphere of the potato, possibly providing long-term protection to the infection. Therefore, to date, there are numerous studies that demonstrate the ability of the genus *Trichoderma* to effectively control plant-parasitic nematodes through direct interaction.

#### Mycorrhizal Fungi

Strictly direct mechanisms of mycorrhizal fungi against nematodes are not yet described as they normally act through the plant host, either providing the plant with higher nutrient and water uptake, altering root morphology by increasing root growth and branching, or making the plants more competitive for nutrients and space with other plants, or altered rhizosphere interactions (Schouteden et al., 2015; Wani et al., 2017). Recent studies have confirmed these mechanisms, for example, mycorrhizas (*Rhizophagus intraradices* and *Funneliformis mosseae*) reduce tomato root penetration by false root-knot nematode *Nacobbus aberrans* (Marro et al., 2018), in the same way as the application of *Glomus intraradices*, *G. mosseae*, and *G. etunicatum* against *M. javanica* in peach trees (Calvet et al., 2001). In contrast, the increment in root colonization by mycorrhizae (*Rhizophagus clarus, Claroideoglomus etunicatum, Gigaspora rosea, G. margarita, Scutellospora calospora*, and *S. heterogama*) caused an increase in the population of nematodes *Pratylenchus brachyurus* in maize crop (Brito et al., 2018) which is opposite to the effect in cotton (Ferreira et al., 2018). All these mechanisms and their effectiveness on the populations and the capacity of infection of the phytoparasitic nematodes will depend closely on the local environmental conditions. In this respect, alterations of potassium, phosphorus and moisture are the main factors negatively involved in the beneficial effect caused by mycorrhizal fungal (Ferreira et al., 2018). Despite some contradictory reports, the use of mycorrhizal fungi for the biological control of plant-parasitic nematodes has been widely studied in numerous crops, as maize (Alvarado-Herrejón et al., 2019), and even in energy crops such as switchgrass (*Panicum virgatum*) and miscanthus (Miscanthus × giganteus; Emery et al., 2017).

#### Endophytic Fungi

Regarding the nematode-control by endophytic fungi, there is still much speculation on the specific mechanisms by which these fungi antagonize nematodes in most cases, but they are likely quite diverse. Endophytic fungi can directly attack, kill, immobilize, or repel nematodes, confuse them when finding their host, interfere with nurse cell development, compete for resources, or employ a combination of those options (Schouten, 2016). For example, it has been observed how *Acremonium implicatum* can colonize the xylem of tomato roots and, in the soil, parasitize and destroy the eggs of *M. incognita* (Yao et al., 2015). Another example is *F. oxysporum* isolated from banana (*Musa* spp.) that paralyzes and kills the root-lesion nematode (*Pratylenchus goodeyi*; Mwaura et al., 2010). They can also produce nematicidal secondary metabolites, as e.g., chaetoglobosin A, chaetoglobosin B, flavipin, 3-methoxyepicoccone and 4,5,6-trihydroxy-7-methylphthalide produced by *Chaetomium globosum* against *M. incognita* (Khan et al., 2019), VOCs against *M. javanica* produced by *Daldinia cf*. *concentrica* (Liarzi et al., 2016), or the production of alternariol 9-methyl ether by *Alternaria* sp. against the pinewood nematode or pine wilt nematode *Bursaphelenchus xylophilus* (Lou et al., 2016). Only a few reports show opposite results, such as the application of entomopathogenic fungus, e.g., *B. bassiana* that increased potato nematodes reproduction and potato tubers damage caused by *Ditylenchus destructor* and *D. dipsaci* (Mwaura et al., 2017).

### Filamentous Fungi From the Genus *Trichoderma*, Mycorrhizal and Endophytic Fungi as Systemic Resistance Inducers for Nematode Control

#### Genus *Trichoderma*

Within their capacities as BCAs, it is worth highlighting the ability of filamentous fungi to stimulate plant defenses against pathogens. Calderón et al. (1993) showed for the first time, the induction of ISR mediated by *Trichoderma* in the grapevine, and one of the first clear demonstrations of induced resistance by *Trichoderma* showed that the treatment of soil with *T. harzianum* improved the resistance of bean plants to diseases caused by fungal pathogens *Botrytis cinerea* and *Colletotrichum lindemuthianu* (Bigirimana et al., 1997). Subsequently, numerous studies have shown that, by colonizing the plant roots, *Trichoderma* stimulates their defense mechanisms against numerous phytopathogenic microorganisms, including nematodes (Hermosa et al., 2012). Numerous attempts have been made to control plant-parasitic nematodes with this genus. *Trichoderma* is capable of inducing resistance in a wide variety of plant species, which leads to transcriptomic, proteomic and metabolomic modifications in the plant (Mukherjee et al., 2012). This systemic defense stimulation prepares the immune response of the plant, allowing a faster response after the priming against the subsequent attack of any kind of pathogen and thus reducing the possibility of disease spread (Hermosa et al., 2012; Bisen et al., 2016; Mendoza-Mendoza et al., 2018). In most cases, this ISR is regulated by a JA/ET signaling, as described by Leonetti et al. (2014). They observed that SA signaling is down-regulated in the early stages of *M. javanica* infection in tomato roots while, the response mediated by JA/ET is induced in tomato roots treated with the fungus, which indicates that the presence of *Trichoderma* activates the ISR within the plant. However, it has recently been described that the SA pathway also participates actively in this regulation (Martínez-Medina et al., 2017a, b). Similarly, the induction of defenses in tomato against *F. oxysporum* f. sp. *lycopersici* by *T. virens* is also, mediated through both JA and SA (Jogaiah et al., 2018). Numerous studies have shown how both hormones coordinate the resistance and susceptibility of plants to nematodes (Martínez-Medina et al., 2017a, b). In this respect, the root-knot nematode-plant interaction is highly dynamic, as plant responses differ significantly between the initial stages and the latest infection stages. Yet, the induction of defenses by *Trichoderma* is a plastic phenomenon. In the first phase, the presence of *T. harzianum* stimulates faster SA-mediated defense responses, to protect the roots against nematode invasion. In the second phase, when *M. incognita* suppresses JA-related defenses in the roots, *Trichoderma* stimulates the expression of JA-dependent defenses, thus antagonizing the suppression of defenses mediated by *M. incognita*, which leads to a reduction in development and reproduction of the nematodes. Once parasitism is established, the fungus increases the activation of SA-dependent defenses probably through the recognition of eggs, which can contribute to improving defenses against the invasion of new juveniles (Martínez-Medina et al., 2017b). Recently, it was proved not only the ability of *T. atroviride* to induce systemic resistance in tomato plants against the nematode *M. javanica*, but its heritability, as the offspring of those plants inoculated with *Trichoderma*, inherited the resistance against *M. javanica* (de Medeiros et al., 2017). This heritability extends to the growth promotion induced by this fungus during its interaction with the plant that is accompanied by a significant reduction in the defenses dependent on the SA and JA pathways. In this respect, it is very interesting to highlight how fungal symbiosis and nematode infection induce epigenetic changes in the plants probably guided by small RNAs (Cabrera et al., 2016; de Medeiros et al., 2017; Medina et al., 2017; Ruiz-Ferrer et al., 2018), that includes changes in plants methylomes (Hewezi et al., 2018). It has been recently demonstrated how epigenetic and post-transcriptional modifications control the interactions between the plants and their surrounding microorganisms, that is, these regulatory mechanisms play a key role in promoting plant resistance to pathogens facilitating the establishment of symbiotic relationships. Therefore, it is important to consider the role of these modifications in the adaptation of the plant to environmental stress, including the resistance of the plant to the pathogens and the formation of symbiotic relationships, as well as in the study of the interaction between plant-pathogen-beneficial microorganism, especially because symbiosis and pathogenesis share similar signaling mechanisms (Zogli and Libault, 2017; De Palma et al., 2019). Furthermore, it has been recently described that commercial formulates of some *Trichoderma* spp. strains induced resistance to *M. incognita* in tomato in split-root system experiments and additionally, an additive effect to that of the tomato *Mi 1.2* resistance gene was also observed (Pocurull et al., 2020). It opens possibilities for integrated pest management of root-knot nematodes with BCAs and other combined control methods.

The high-inoculum dose of *Trichoderma* can also trigger a SA-mediated SAR response similar to that caused by necrotrophic pathogens (Mukherjee et al., 2012; Bisen et al., 2016); hence, the defense induced by this genus can be mediated by ISR or SAR pathways and also by a complex signaling network that connects the SAR and ISR (Mendoza-Mendoza et al., 2018). Therefore, the induction of the SA, ET and JA pathways in the same plant when colonized by *Trichoderma* suggest the presence of alternative ISR pathways and a complex signaling network that connects the SAR and ISR defense response pathways. This varies upon the plant species, the *Trichoderma* strain and the pathogen against which the defense response is directed (Brotman et al., 2012; Nawrocka and Małolepsza, 2013).

#### Mycorrhizal Fungi

The control of mycorrhyzal symbiosis is a finely tuned process that involves multiple regulatory components functioning at different levels. Research over the past few years revealed the critical roles of defense phytohormones in modulating mycorrhizal interactions, from early recognition/colonization events up to the final arbuscular formation and degradation (Liao et al., 2018). The ability of mycorrhizal fungi to activate ISR in the plant against the possible attack of pathogens and/or pests has been reviewed by numerous authors, highlighting Pozo and Azcón-Aguilar (2007), Hohmann and Messmer (2017), and Jacott et al. (2017). Mycorrhizal fungi initially trigger plant defense mechanisms similar to a biotrophic pathogen, but then modulate plant responses for successful colonization. In this sense, SA is known to activate responses against biotrophic pathogens, and it is enhanced by mycorrhizal fungi, although, it is subsequently downregulated, which finally allows the symbiotic interaction. Once the symbiosis is established, the microbe-induced resistance and priming regulated by JA is activated, similarly to the responses controlled by JA and ET pathways against necrotrophic pathogens (Hohmann and Messmer, 2017).

Plant resistance by mycorrhizal fungi against phytopathogenic nematodes have been described, however, in conventional pathogenic-assays, it is difficult to distinguish to what extent the decrease in infection is due solely to systemic resistance or to a direct effect. To try to determine the ability of mycorrhiza induced resistance against nematodes a good strategy is the use of split-root methodology. In this respect, Vos et al. (2012a) showed how the inoculation of tomato roots with *F. mosseae* in one of the compartments reduced the infection rates of *M. incognita* and *Pratylenchus penetrans* in the compartment without fungus, through altered root exudation. The mechanisms through which this increase in the systemic defensive capacity of the roots occurs are related to the activation of genes that encode chitinases, PR proteins, enzymes involved in the detoxification of ROS (whose accumulation occurs during hypertrophy and death cell caused by nematodes) such as glutathione S-transferase or the superoxide dismutase (SOD), enzymes involved in the lignin biosynthesis, and in the shikimate pathway which in turn, produces precursors of various aromatic secondary metabolites against nematodes (Schouteden et al., 2015; Sharma and Sharma, 2017b; Balestrini et al., 2019).

Concerning hormones not directly related to plant defensive responses, it is known that strigolactones, after being exuded from the root, activate hyphal branching and enhanced growth and energy metabolism of symbiotic AMF, and once the symbiosis is established, its production by the plant roots fades (Rochange et al., 2019). Regarding the nematodes, it has been proven that the strigolactones do not contribute to cyst nematode (*Heterodera schachtii*) hatching but they do play a role in host attraction and subsequent invasion (Martinez et al., 2019). In rice, signaling mediated by strigolactones suppresses jasmonate accumulation and promotes RKN infection (Lahari et al., 2019). In contrast, strigolactones play a positive role in nematode defense in tomato (Xu et al., 2018). Although, further research needs to be undertaken, another way to modulate plant responses by mycorrhization could be altering strigolactones -plants production, which might influence plant-nematode interaction.

Regarding the ECMF, colonization of pines (*Pinus thunbergii*) promoted a lasting SAR against the pine wilt nematode *Bursaphelenchus xylophilus*, which is transmitted by beetles of the genus *Monochamus* and feeds by colonizing the vascular bundles (Nakashima et al., 2016). Interestingly, those fungi do not only act systemically controlling nematodes-infection but enhance plant defenses against pathogens transmitted by the nematodes, as it is the case with the Grapevine Fanleaf Virus (GFLV) transmitted by the nematode vector *Xiphinema index*, against which AMF *R. intraradices* induces systemic protection (Hao et al., 2018).

Other examples of systemic resistance produced by AMF are a reduced nematode infection, due to the activity of phenolics and defensive plant enzymes, i.e., peroxidase (PO), polyphenol oxidase (PPO), and SOD, together with a significant reduction of malondialdehyde (MDA) and hydrogen peroxide (H_2_O_2_) content in tomato roots inoculated with *R. irregularis*, that also enhanced plant growth (Sharma and Sharma, 2017a). Similarly, the application of *G. mosseae* using a split-root system against the sedentary nematode *M. incognita* and the migratory nematode *P. penetrans*, in tomato indicated that the infection by both different types of nematodes can be controlled by AMF, but only systemically (Vos et al., 2012b). Similar results were observed in banana (*Musa* spp.) with split-roots using AMF *Glomus intraradices* against the migratory nematodes *Radopholus similis* and *Pratylenchus coffeae* (Elsen et al., 2008). In other cases, it is not clear whether ISR or a direct mechanism are acting against nematodes or even both mechanisms simultaneously. One example is the reduction of *P. penetrans* infestation in apple seedlings by AMF (Ceustermans et al., 2018), and of *Meloidogyne arenaria* in red ginger (*Alpinia purpurata*) by *Gigaspora albida*, *Claroideoglomus etunicatum*, and *Acaulospora longula* (Da Silva-Campos et al., 2017), or the control of the migratory endoparasitic nematode *Scutellonema bradys* in yam (*Dioscorea* spp.) by *F. mosseae* and *Glomus dussii* (Tchabi et al., 2016). In contrast, Frew et al. (2018) described that the root application of *Claroideoglomus entunicatum*, *Funneliformis coronatum*, *R. irregularis*, and *F. mosseae* in wheat produced an increase of the plant-parasitic nematode *Pratylenchus neglectus* populations. This fact is due to a decrease of root benzoxazinoid glucoside accumulation, an important defense metabolite against the nematode, probably necessary for the root colonization of the fungus itself.

#### Endophytic Fungi

The split root system methodology was also used to study systemic resistance induced by endophytic fungi, as *F. oxysporum* against the root-knot nematode *M. incognita* in *A. thaliana* (Martinuz et al., 2015), in tomato (Dababat and Sikora, 2007), and in banana against the burrowing nematode *Radopholus simili* (Vu et al., 2006). Another endophytic fungi, *Fusarium moniliforme* also ISR toward *Meloidogyne graminicola* in rice (Le et al., 2016). *Fusarium* spp. achieve this systemic induction due to the synthesis and release of chemical compounds such as 4-hydroxybenzoicacid, indole-3-acetic acid (IAA) and gibepyrone D, which, in addition to being toxic directly to the nematodes, induce defense mechanisms against nematodes in plants (Bogner et al., 2017). But endophytic fungi act also systemically causing the synthesis and transport of chemical defense components in the plant. This is the case of the secondary metabolite forskolin synthesized in the medicinal plant *Coleus forskohlii* after being inoculated with the endophytic fungi of the stem *Phialemoniopsis cornearis* and *Macrophomina pseudophaseolina* and radicular *Fusarium redolens*, as they enhance the expression of diterpene synthases, that enhances tolerance to *M. incognita* (Mastan et al., 2019). The ability of *P. indica* to modify plant stress-hormones has been extensively studied (Le-Xu et al., 2018). Although, several studies show that the application of this fungus against *M. incognita* (Varkey et al., 2018) and *Heterodera glycines* (Bajaj et al., 2015) reduces the incidence of the disease and improves plant growth, the exact role of systemic resistance is still unsolved. Similarly, *Phialemonium inflatum* in cotton against *M. incognita* (Zhou et al., 2018), *Nigrospora* sp. in sengon plant (*Paraserianthes falcataria*) against *Meloidogyne* spp. (Amin, 2015), *Penicillium brefeldianum* in melon against *M. incognita* (Miao et al., 2019) or *Fusarium solani* and *F. oxysporum* in tomato against *M. incognita* (Bogner et al., 2016) were described.

*Pochonia chlamydosporia* is a nematophagous fungus, but it is also a root endophyte that can colonize the roots of higher plants due to a partial suppression of defensive responses mediated by JA. They also reduce the flowering time, stimulate plant growth and increase seed production in *A. thaliana* (Zavala-González et al., 2017) and a greater root colonization was directly related to a lower incidence of *M. javanica* in tomato roots (Escudero et al., 2017). *P. chlamydosporia* induces systemic resistance against *M. javanica* in tomato (but not in cucumber) by activating a defensive response mediated by SA (Ghahremani et al., 2019). Interestingly, in the absence of nematodes, it is observed how it promotes systemic resistance mediated by JA in barley (Larriba et al., 2015). Another example in forest plant-species is that the presence of the endophytic fungi *Gaeumannomyces cylindrosporus*, *Paraphoma chrysanthemicola*, *Phialophora mustea*, *Exophiala salmonis* and *Cladosporium cladosporioides* modifies the systemic defensive responses reducing the incidence of the nematode *Bursaphelenchus xylophilus* in pine (Chu et al., 2019). On the other side, the attack of the nematodes can systematically alter the defenses of the plant and prevent its colonization by endophytic fungi. This has been observed in *Pinus tabulaeformis* after the infection of the pinewood nematode (*Bursaphelenchus xylophilus*) where the diversity of endophytic fungi in the roots was reduced to about 80% (Chu et al., 2018).

In the same way as for mycorrhizal fungi, strigolactones were necessary for the establishment of the beneficial interaction, at least in the case of the endophytic fungus *Mucor* sp. on *A. thaliana* roots. Once the symbiosis is established, the fungi decompose strigolactones *in planta* and downregulate the expression of strigolactones biosynthesis genes (Rozpądek et al., 2018), preventing, therefore, root invasion by nematodes (Martinez et al., 2019).

## Conclusion

**FIGURE 1:**
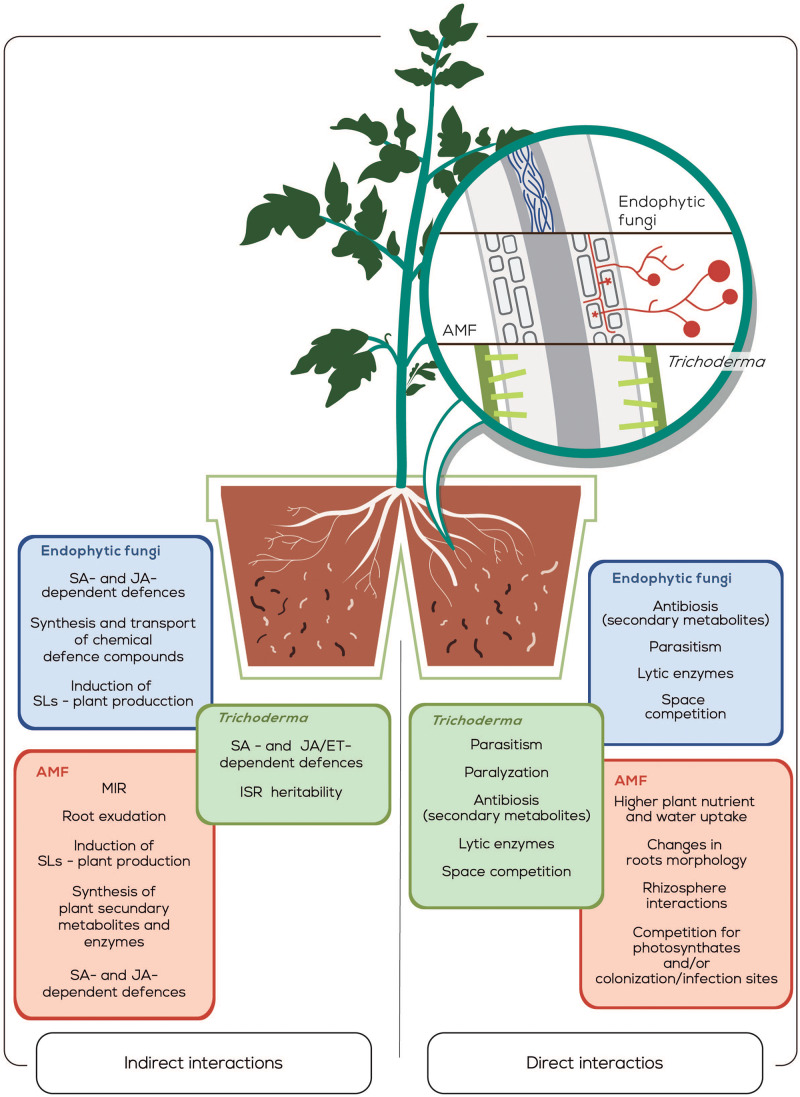
Graphical representation of direct and induced resistance against plant-parasitic nematodes by filamentous fungi in a split-root system. The different tissue colonization strategies used by the three fungal groups are schematically represented in the microscopic enlargement of the roots. The mechanisms of fungus to induce resistance in plants either by direct or indirect interaction strategies against nematodes are indicated in colored boxes. The effects of the filamentous fungi on nematodes basically are: To increase J2 mortality, to decrease the hatching and/or nematodes infection rate, as well as to alter nematodes development inside the plant and/or their reproduction. Abbreviations correspond to arbuscular mycorrhizal fungi (AMF), salicylic acid (SA), jasmonic acid (JA), systemic acquired resistance (SAR), induced systemic resistance (ISR), strigolactones (SLs), and mycorrhizal induced resistance (MIR).

Biocontrol strategies for plant-parasitic-nematodes constitute a valid alternative to toxic chemical nematicides. Thereby, a wide diversity of effective strategies based on the use of filamentous fungi used as BCAs are described. They work through two main kinds of mechanisms of action, i.e., those that include the production of secondary metabolites (antibiosis), lytic enzymes, and space competition by *Trichoderma*. AMF directly acts providing higher nutrient and water uptake to the plant, modifying root morphology and altering the rhizosphere interactions, or competing for photosynthates or colonization/infection sites. Endophytic fungi reduce the attack of the plant-parasitic nematodes by parasitism, by paralyzing the nematodes, through antibiosis, by lytic enzymes production and also by space competition. The second group of action mechanisms are the induction of plant defenses, such as the activation of SAR and ISR by *Trichoderma*, which seems also heritable. As well as, the modification of roots exudates, strigolactones production, plant secondary metabolites and enzymes production by AMF. Finally, the induction of SAR and ISR, the transport of chemical defense components through the plant and the strigolactones production by endophytic fungi (Figure 1).

The scenario in agricultural systems is very complex as different microorganisms of the rhizosphere and plant-species are present; thus they may respond differently to BCAs. The integration of all microorganisms and plant-responses will determine the final output after the BCA treatment as it is a multifactorial response. Additionally, the strong influence of the local environment that for example, highly determine endophytic communities, is another complexity level to include in the agricultural systems. Therefore, BCAs can have variable control ability when established in different soils, and their success can be difficult to predict. However, there are commercial formulates with some of those filamentous fungi mentioned, effective for nematodes control in experimental conditions for particular crops, but scarce scientific information is still available of experiments on the field. The integration of empirical data knowledge in the field after the treatment with a particular fungi strain or formulate, together with detailed analysis of the plant responses at the molecular level in a particular crop could help to a deep understanding of those complex interactions. Yet, holistic approaches for soil-rhizosphere microbiota detection and characterization of specific plant responses could assist in identifying and predicting major problems as the presence of BCA antagonist in the soil or changes in the rhizosphere biota that may influence the final output. This would allow better practices for an effective nematode-control on a particular crop. Moreover, an in-depth knowledge on the molecular mechanisms of induced resistance by BCAs could allow perhaps to manipulate the plants directly, however, synergic effects derived for example of secondary metabolites induced also by some BCAs should also be integrated. Another question is that usually, BCAs based control methods are slow to implement as they may need time to increase their population up to the level required to be effective for PPN control. Yet, the growers need alternative control methods during this period. The durability of those BCA-based techniques is also a great interrogate because of the mentioned complexity of the agricultural systems.

Perhaps, a feasible scenario would be to design integrated pest management based first on a well stablished method on a ground base, such as those chemically based, and slowly to introduce BCAs together with a detailed monitoring plan with the intention to gradually decrease the dependence on chemical control. We should not also discard the possible implementation of other complementary alternative control methods, including those based on host plant genotypes that may have an enhanced response in combination with BCAs.

In summary, the use of filamentous fungi of the genus *Trichoderma*, mycorrhizal and endophytic fungi as BCAs is a promising durable biocontrol strategy in agriculture against plant-parasitic nematodes (Figure 1) due mainly to the wide diversity of mechanisms of action described above, that in most of the cases act also in combination. However, there are still major question to address with further research. Moreover, recent reports point to a heritable biocontrol after BCAs infection possibly driven by still not well-known epigenetic mechanisms, opening a new field of research with special applied interest.

## Author Contributions

JP and PA-U contributed to the search for information and references in different databases, they gathered most of the peer-reviewed manuscript used on this topic, and highly contributed to the manuscript writing. CE contributed to the manuscript writing and the correction and critical reading, as well as to the knowledge on the nematode field.

## Conflict of Interest

The authors declare that the research was conducted in the absence of any commercial or financial relationships that could be construed as a potential conflict of interest.
